# Medical Intelligence

**Published:** 1819-07-01

**Authors:** 


### MEDICAL INTELLIGENCE.

### 1. *Hunterian Society.*

A Society has been established in London, bearing the designation of the "*Hunterian Society.*" It professes the most friendly feelings towards all similar existing institutions, and is founded principally, but not exclusively, for the accommodation and benefit of medical men residing in the eastern parts of the metropolis.

Its objects are to concentrate the zeal and experience of a large number of respectable practitioners, whose places of residence are at a distance from professional associations already existing; and to receive and discuss communications on medical and surgical subjects.

### 2. *Widows of Army Medical Officers.*

The Annual Meeting of the Society for the Benefit of Widows of Officers of the Army Medical Department took place a few days since. The Funds of this excellent Society, established only four years ago, have accumulated far beyond the most sanguine expectations of its original Founders. The number of Subscribers are upwards of five hundred and eighty, whose Contributions, added to the Dividends on a Capital of between thirteen and fourteen thousand 3 per Cent. Stock, produce an Annual Income of nearly TWO THOUSAND POUNDS.

The friends of this Institution, amounting to nearly eighty Gentlemen, afterwards dined together at the Thatched House Tavern, St. James's Street; amongst whom were associated, Dr. Baillie, Sir Gilbert Blane, Bart. Sir Everard Home, Bart. and several other distinguished members of the medical profession, both civil and military.

3. A regular Establishment having been formed by Government for the exclusive Treatment of Diseases of the Eye, it becomes the duty of the individual entrusted with its superintendance, to render the Institution as useful to the Public as possible. Sir William Adams, therefore, proposes to deliver Annually a Course of Lectures upon Ophthalmic Surgery, in which his peculiar operations and modes of practice will be fully detailed. And as the Cases which occur in the Public Establishment, are more particularly of the *chronic* kind, it is his intention immediately to establish a Dispensary in the same neighbourhood, for the reception of the Poor in civil life, who labour under the *acute* forms of disease. The opportunities of illustration afforded by these Lectures, conjoined with the operative and practical parts of the science in these two Institutions, will it is hoped, constitute a complete school for Ophthalmic Surgery. To these Lectures, and to the Practice of the two Institutions, the medical officers of the three branches of the Public service will be admitted gratis.

4. Dr. JOHN B. DAVIS, Licentiate of the Royal College of Physicians of London; Physician of the Universal Dispensary for Children; Senior Physician of the London Dispensary; and Physician of the Surrey Dispensary, &c. &c. will commence his next Course of Lectures on that Branch of the Practice of Medicine which relates to the Diseases, and Medicinal Management of Children, and young Persons; on the first Thursday of July, at the Universal Dispensary for Children, No. 5, St. Andrew's Hill, Doctors' Commons.

### *To the Editor of the Medico-Chirurgical Journal.*

SIR,

The committee of "Associated Apothecaries and Surgeon-Apothecaries of England and Wales," being deputed at their last annual meeting in July, 1818, to prepare certain Rules, &c. for the organization of that Society, beg leave to offer the following, for the consideration of the Profession, previous to their being proposed for confirmation at the next annual meeting, which will be held as usual at the Crown and Anchor Tavern, Strand, on Wednesday, July the 21st, 1819.

As a most extensive medium of medical communication, I shall feel obliged by your introduction of them into the next number of your very valuable and widely circulating publication.

I remain, Sir,

Your most obedient Servant,

JAMES PARKINSON,

Chairman to the Committee.

*Hoxton Square, June 12, 1819.*

---

### *Rules and Regulations of the Associated Apothecaries and Surgeon-Apothecaries of England and Wales.* 1819.

### SECTION I. OF ITS NAME.

THIS Association shall in future be called, "*The Associated Apothecaries and Surgeon-Apothecaries of England and Wales.*"

### SECTION II. OF ITS OBJECT.

ART. I. It is constituted to promote the improvement of those branches of the profession exercised by general practitioners, and by thus supporting their respectability, to protect in the most effectual manner the interests of the community at large.

II. To ensure this respectability, by embracing every opportunity of securing to the practice of surgery and of midwifery, similar regulations to those which now legally control the practice of medicine.

III. To endeavour to prevent ignorant and unqualified persons from practising medicine, by watching over the operations of the apothecaries' act, and by communicating to the society of apothecaries all cases in which it shall be discovered that the Regulations of that act shall have been infringed.

IV. To form and preserve lists of practitioners legally appointed; and thus, by distinctly separating the two classes, to enable the public, in some degree, to distinguish the fair and authorized practitioner from the ignorant unqualified pretender.

### SECTION III. OF MEMBERS.

I. This association shall consist of the hereinafter mentioned gentlemen, subscribers to the original association.

II. The requisite qualification for the admission of every future member shall be, that he is legally authorized to act as an apothecary or a surgeon.

III. Before admission, a certificate testifying his qualifications, signed by two members of this association, and accompanied by a declaration of the candidate's wish to become a member, shall be transmitted to the chairman of the metropolitan general committee, or to the chairman of the county general committee, as the case may be, at least one fortnight before the ordinary meetings of such committee, and the election shall take place at the next quarterly meeting of the association.

IV. The fee for admission shall be one guinea, to be paid on admission

V. That all members present at the determination of any question, shall possess an equal right to vote.

### Section IV. OF MEETINGS.

I. The association shall meet for general purposes once in each year, on the third Wednesday in June.

II. One fortnight's notice shall be given of the annual general meeting, in the London medical journals, and by advertisements in two morning and two evening papers.

III. The association shall on that occasion assemble at one o'clock in the afternoon. The president shall take the chair for the dispatch of business precisely at two o'clock, and the business shall be conducted in the following order; viz. Candidates proposed. Candidates ballotted for. Minutes of the preceding meeting read and discussed. Report of the general committee received, read, and considered. Miscellaneous business, including the revisal of the laws, and whatever may relate to the general management of the association.

IV. No question relative to the alteration of any fundamental laws of the association, shall be discussed, unless notice of it, signed by at least three members, shall have been transmitted to the president, at least three months before the annual meeting of the association; so as to allow time for country members being duly apprized of such intention.

V. All questions on which a difference of opinion may exist, shall be decided by a show of hands, or division, at the option of the members present.

VI. In cases where the suffrages are equal, the president shall have a casting vote.

VII. After the conclusion of the business of the general meeting, the members shall dine together, at five o'clock.

VIII. Special general meetings of the association may be called by the president, by virtue of his office; and shall be summoned by him, on receiving a requisition signed by five members of the general committee, or by fifteen members of the association; the proposed object of such meeting being announced to the members in each case.

IX. Special general meetings of the association, shall be subject to all the foregoing regulations, except article VII.

X. Members residing in the country shall not only have the right of attending all meetings of the association, but their presence will always be considered as desirable, particularly at the annual general meeting.

### Section V. OF COMMITTEES.

I. A general committee shall be formed, to whom shall be entrusted the conducting of the business of this association, between its general meetings, according to the existing laws.

II. The general committee shall consist of the president of the association and twenty-four members, twelve of whom shall be chosen from such members of this association as do, and twelve from such as do not belong to the Apothecaries' Society.

III. The president shall be chairman of the general committee, and have a casting vote.

IV. One half of each class of the committee shall go out every year by rotation; such members shall nevertheless be eligible for re-election.

V. The general committee shall meet quarterly for dispatch of business, viz. on the first Monday in September, December, March, and June.

VI. Meetings of the general committee may be called by the chairman, by virtue of his office, and shall be summoned by him, on receiving a requisition, signed by five members of the committee.

VII. The general committee shall appoint a sub-committee, out of its own members, whenever it shall be deemed necessary.

VIII. At meetings of the general committee, five members shall form a Quorum.

IX. At meetings of the sub-committee, three members shall form a quorum.

X. The general committee shall present a report of its proceedings during the last year, to the association, at its annual meeting in June.

XI. The members of this association who reside in the country, shall form themselves into county committees.

XII. Each county committee, when duly organized, shall form sub-committees for as many districts as the extent and population of the county may, after mature consideration, appear to be requisite.

XIII. The county and district committees shall choose from their own bodies respectively, a chairman and a secretary.

XIV. The meetings of the county and district committees shall be held at periods and places most convenient to the members of such county or district.

XV. A general meeting of the members in each county shall be held every year, at least one month previous to the annual general meeting in the metropolis: the day and the place of meeting to be determined by the county committee.

XVI. The business of the annual county meeting shall be to receive communications from the several district committees, to receive application for the admission of members, and to transmit the same to the metropolitan committee, with such other information, and such suggestions, as may appear to be of sufficient importance to require the attention of the general meeting.

XVII. The objects of the county and district committees shall be, to take cognizance of, and communicate, any instances of infringement upon the regulations of the apothecaries' act in their respective neighbourhoods; and to inquire into all cases of gross ignorance, or impropriety of conduct; and to transmit the same through their chairman (when duly authenticated) to the chairman of the metropolitan committee for the use of the association; and, if necessary, for further transmission to the society of apothecaries.

### Section VI. OF OFFICERS.

I. The officers of the association shall consist of a president, two vice-presidents, three treasurers, and a secretary.

II. The president and vice-presidents shall be chosen annually.

III. There shall be two vice presidents, one only of whom shall be chosen from the apothecaries' society.

### Section VII. OF THE PRESIDENT.

It shall be the province of the president to preside at the meetings of the association, and to enforce an observance of its rules.

### Section VIII. OF THE VICE-PRESIDENTS.

In the absence of the president, his duties shall devolve upon one of the vice-presidents present.

### Section IX. OF THE TREASURERS.

I. It shall be the duty of the treasurers, to invest and keep in public securities, in their joint names, all monies belonging to the association (except such balance as shall be necessary to defray current expenses), for the exclusive use and benefit of the association.

II. The signatures of two of the Treasurers shall be necessary to the disposal of any part of the funds of the association.

### Section X. OF THE SECRETARY.

It shall be the office of the Secretary to attend the several meetings of the association in London; to enter on the journals the minutes of the proceedings of such meetings; to keep a printed list of the members of the association; and to act under the general direction of the president.

*Mem.* The general meeting of this society will be on the third Wednesday (21st) of July, at one o'clock, at the Crown and Anchor Tavern in the Strand, London. Dinner, at five o'clock on the same afternoon, will be provided for such gentlemen as shall signify their intentions of dining with the association, to Mr. OTTLEY, of the Crown and Anchor, three days previous to that of meeting.

